# Editorial: Aging epigenome and longevity

**DOI:** 10.3389/fragi.2025.1739913

**Published:** 2025-11-14

**Authors:** Sandeep Kumar Singh, Burkhard Poeggeler

**Affiliations:** 1 Department of Medical Biotechnology, All India Institute of Medical Sciences, Nagpur, India; 2 Department of Physiology, Johann-Friedrich-Blumenbach-Institute for Zoology and Anthropology, Faculty of Biology Georg August University Göttingen, Göttingen, Germany

**Keywords:** aging, neurodegenaration, epigentic alteration, longevity, mitochondrial dysfunction (MD)

The Research Topic “*Aging Epigenome and Longevity*” brings together a Research Topic of innovative research that deepens our understanding of how epigenetic processes shape human aging and longevity. Through four distinct articles, this Research Topic offers readers fresh insights into the many ways our genes, environment, and lifestyle come together to influence health as we age. From population-based studies to new methodological advances and detailed molecular investigations, these contributions showcase the rapid progress and exciting possibilities in the field of aging epigenetics.

Chen et al. original study shines a light on the LEF1 gene, revealing how its expression drops with age due to DNA methylation. Using both transcriptomic data and cell models, the authors show that as the promoter region of LEF1 becomes more methylated, the gene’s activity declines, promoting inflammation and reactive oxygen species production in immune and brain cells. These experiments highlight how a single epigenetic change can ripple through important biological pathways, linking LEF1 not only to healthy aging but also to diseases driven by inflammation and neurodegeneration. In doing so, this work points to both LEF1 as a promising biomarker and to epigenetic interventions as potential future therapies.

Martínez-Enguita et al. take a different tack, applying the power of neural networks to DNA methylation patterns. Their NCAE-CombClock model sets a new standard for precision in estimating biological age by fusing machine learning-derived features with established CpG markers. This approach does more than just estimate age: it uncovers methylation signatures tied to processes like neural development, immune function, and metabolism, even in younger populations. The research gains extra relevance through its application to pediatric Crohn’s disease, demonstrating how the aging process becomes visible even in the trajectories of childhood illness. Such models move the field closer to providing personalized aging insights that could 1 day guide individualized interventions.

Looking broadly at the rapidly expanding toolkit for measuring biological age, Mathur et al. provide a comprehensive review of approaches old and new. Their synthesis of findings from 140 studies covers everything from traditional telomere length and epigenetic clocks to newer markers involving exosomes, stem cells, and even the gut microbiome. Their critical review stresses the complex relationship between the classical “hallmarks of aging” like genomic instability and mitochondrial dysfunction and our ability to actually measure and influence healthspan. By highlighting both the strengths and limitations of current methods, this article guides the field toward more reliable, multidimensional measures suited for real-world application.

Kim et al. focus on the performance of epigenetic clocks in a South Korean population, making important distinctions between “first-generation” clocks (built to estimate chronological age) and “second-generation” clocks (designed for biological age and health prediction). Their findings reinforce that newer clocks are better at tracking health outcomes ranging from disease risk to lung function and lifestyle factors while noting that their accuracy can differ by ethnicity and environment. Notably, their research emphasizes the role of lifestyle factors such as body mass, smoking, alcohol use, and physical activity in accelerating or slowing the biological aging process.

Together, these articles make it clear that aging is a complex process, governed by genetics, environment, and chance—all acting through the flexible landscape of the epigenome. They show that biological age, as measured by new tools and techniques, can deviate significantly from the number of our birthdays, offering clinicians and researchers better ways to track disease risk and functional decline. The inclusion of machine learning, critical large-scale reviews, and population-specific research points to the next chapter for the field: developing standardized, interpretable, and broadly applicable measures that guide interventions for longer, healthier lives.

Bringing these threads together, the Research Topic paints a hopeful picture. Through high-resolution biomarkers, artificial intelligence, and careful validation across diverse groups, researchers are poised to decode the mysteries of the aging epigenome. Such insights will be essential as we work toward interventions that not only extend life but also preserve quality of life.

